# Practical Hints upon Medical Examination for Life Assurance.—IV

**Published:** 1908-09-12

**Authors:** William P. S. Branson

**Affiliations:** Junior Medical Officer, Sun Life Office.


### Life Assurance.

### PRACTICAL HINTS UPON MEDICAL EXAMINATION FOR LIFE ASSURANCE—IV.

By WILLIAM P. S. BRANSON, M.D., M.R.C.P., Junior Medical Officer, Sun Life Office.

### The Lungs.

The detection of past or present pulmonary tuberculosis is perhaps the most important function of the medical referee. He will of course be guided in making his examination by such points as the age and physique of the candidate, his family record, and any suggestive items of illness in the past history, conducting his examination of the lungs with a rigour proportionate to the probabilities in any given case. But if the detection of this malady be difficult for the clinical practitioner it is doubly so for the life-office examiner, since *ex hypothesi* the disease is unlikely to be active at the time the proposer presents himself. Assuming then that there is something about a case which prompts the suspicion of a tuberculous constitution, it becomes the examiner to investigate the lungs with particular attention, especially as regards their upper limits. In a few instances he may discover frank evidences of a past lesion, as for example an impairment of resonance, or a visible increase in the depth of the supra- and infra-clavicular hollows on one side or the other. But this is far from common. For the most part he will find himself dependent for information upon auscultation only, an abnormal harshness of breath-sounds, an occasional click or crepitation at one or other apex. In such an examination as this, when the attention is strained for the appreciation of an intrinsically insignificant sound, and judgment is perhaps a little biassed by suspicious circumstances in the family history, past illnesses, or physique of the examinee, there are two traps worthy of notice and caution. The first depends upon the fact that during normal respiration a number of vesicles, particularly along the anterior borders of the upper lobes, are functionless. These temporarily inactive vesicles are expanded by the first deep inspiration taken after a period of quiet breathing, and their expansion is attended by a sharp crepitant sound. If therefore the stethoscope be applied to the chest during this first deep inspiration, it is likely that crepitations will be heard which are purely physiological. Yet, once heard in a case under suspicion, these sounds are prone to exert an unconscious bias upon the judgment of the examiner and are most inimical to a judicial estimate of the remaining evidence before him. It is therefore a wise practice to instruct the examinee to draw four or five deep breaths before the auscultation of the lungs is commenced. The second trap lies in the ease with which crepitations can be manufactured by the friction of the chest-piece of the stethoscope against the skin. This is particularly the case with the auscultation of the apices of the lungs above the clavicles, for here there is very generally a hollow—and often in the suspicious chests under notice a deep one—rendering it a difficult matter to obtain good apposition of the chest-piece to the skin. Moreover, the skin of this region is under the influence of the platysma myoides muscle, a muscle quite frequently thrown into contraction during the effort of deep inspiration invited by the examiner. This trap is a hard one to avoid. It would seem that some skins are more apt to produce these factitious crepitations than others; in practice it is not the rough skins so much as the greasy ones which prove the most frequent offenders. The composition of the chest-piece is of considerable moment, ivory and metal having, in the opinion of the writer, a definite advantage over ebony or vulcanite. Something further can be done to eliminate error by instructing the proposer to breathe as far as possible without throwing into action the platysma myoides or the muscles of the shoulder girdle, a favourite practice with some examinees and one entailing an undesirable movement of the skin in the supra-clavicular fossa. If these precautions be borne in mind, and the conclusion be drawn that there do in fact exist added sounds of any kind in the apical region of the lung, it may be taken, from the life assurance point of view at least, that there has been a tuberculous lesion of the lung, and the case should be appraised on this assumption. Apart from the matters above detailed, there is little about the examination of the lungs which is in any way special to the examination for life assurance.

### The Urine.

Anomalies of the urine rank with heart-murmurs and the suspicion of tuberculosis in the frequency with which they supply hard problems to the medical authorities of life offices. They deserve therefore a somewhat detailed consideration.

*Albuminuria* is by far the commonest anomaly in the urine of persons apparently healthy. It will not be out of place to offer a few observations upon the relative virtues of the two tests for albumin commonly in use—namely Heller's, or the cold nitric acid test, and that which consists of boiling and then adding acetic acid. It may be said at once that the former is for assurance purposes the more generally useful if it be properly carried out. The vital point in the performance of this test is the attainment of a well-defined line of contact between the urine and the acid. If this be successfully achieved the coagulated albumin is confined to a very narrow section of the column of fluid and becomes obvious even though present in very small quantity. But any diffusion of the two fluids will equally diffuse the opacity produced by the coagulum, and render it correspondingly difficult of detection. This applies of course only to those specimens of urine which, though albuminous, are so only to a minimum degree. The most satisfactory method of carrying out this test, in the writer's experience, is the following:—Half an inch of strong nitric acid is placed in a test-tube; a few drops of the urine are next poured away out of the specimen-glass which contains it, the object of this proceeding being to moisten the lip of the glass, and thus prevent a sudden discharge of the urine when the contact is attempted. This point is one of great importance in practice. The test-tube containing the acid is then inclined so as to be as horizontal as possible, and the lips of the two glasses are brought into apposition. The urine is now slowly poured down the wall of the test-tube, preferably in the track of the acid, which done, the test-tube is gradually, and without shaking, restored to a vertical position and inspected in a good light against a dark background, such as a dark sleeve. The presence of albumin is signified by a band, opalescent or opaque, according to the quantity of albumin present, situated precisely at the line of contact of the two fluids. It is well to wait for a minute or two before accepting the absence of albumin in a given case, since with very small quantities of albumin the reaction may be somewhat slowly established. Haziness and precipitations in the main column of urine above the line of contact may be due to mucus, or urates, or extraneous constituents such as the resin of copaiba when this has been taken by the mouth. These appearances are described as fallacies, but in practice there is no difficulty in distinguishing them from the ring which signifies albumin. A diffuse faint ring at the line of contact is suggestive rather of albumin derived from local irritation of the urethra or bladder than of true albuminuria, for in the latter case the ring of opacity even when faint, is compact and defined.

